# Dissociation between muscle activation, spinal joint load, and subjective perception in passive back support exoskeleton use

**DOI:** 10.1371/journal.pone.0361338

**Published:** 2026-10-26

**Authors:** Hyeonhee Jung, Jangwhan Ahn, Eunsik Choi, Jeongin Moon, Kyoungchul Kong, Jooeun Ahn

**Affiliations:** 1 Biomedical Engineering Research Center, Asan Institute for Life Sciences, Asan Medical Center, Seoul, Republic of Korea; 2 The Lampe Joint Department of Biomedical Engineering, North Carolina State University, Raleigh, North Carolina, United States of America; 3 The Lampe Joint Department of Biomedical Engineering, University of North Carolina at Chapel Hill, Chapel Hill, North Carolina, United States of America; 4 Department of Physical Education, Seoul National University, Seoul, Republic of Korea; 5 Department of Mechanical and Industrial Engineering, University of Massachusetts Lowell, Lowell, Massachusetts, United States of America; 6 Department of Mechanical Engineering, Korea Advanced Institute of Science and Technology, Daejeon, Republic of Korea; 7 Angel Robotics Co, Ltd, Seoul, Republic of Korea; 8 Institute of Sport Science, Seoul National University, Seoul, Republic of Korea; 9 SNU Robotics Institute, Seoul National University, Seoul, Republic of Korea; The Hong Kong Polytechnic University, HONG KONG

## Abstract

One important purpose of back support exoskeletons (BSEs) is to reduce the stress to the lumbar spine and alleviate the low back pain. However, most previous studies introducing BSEs have argued for their effectiveness based only on easily measurable physiological outcomes such as muscle activation. Motivated by this gap, we examined whether the muscle activation of the back muscle during load handling with a passive BSE can faithfully reflect the estimated L5–S1 joint reaction forces and subjective perceptions. Experiments involving 12 healthy young adults revealed that the BSE significantly reduced average thoracic erector spinae muscle activation during both lifting and lowering the load, and lumbar erector spinae muscle activation during lowering. The BSE also reduced integrated thoracic erector spinae muscle activation during lowering. L5–S1 joint reaction forces were also significantly reduced by the BSE (p < 0.001). Participants reported a lower rate of perceived exertion (p < 0.05). However, the lack of associations among all metrics suggests that inferring one measure from another remains limited when evaluating the efficacy of a BSE.

## Introduction

Low back pain (LBP) is the leading cause of disability worldwide [1,2]. Workers in physically demanding jobs, such as manual material handling, face a significantly elevated risk of developing LBP [1]. LBP is associated with physical, mental, and economic deterioration, leading to premature retirement with subsequent mobility impairments and disability [1,3–6]. Although there exists no clear cause-effect relationship explaining the occurrence of LBP, mechanical loading is considered as a primary contributor. Repetitive lifting and bending with excessive spinal loads elevate the risk of intervertebral disc displacement-related outcomes, which are highly associated with LBP in younger adults [7]. To mitigate mechanical load in manual labor tasks, back support exoskeletons (BSEs) have emerged as a promising solution, with ongoing advancements focused on reducing the risk of LBP.

For BSEs to be widely adopted in industrial settings, clear evidence of their efficacy must first be established [8]. Most evaluations have relied on selective metrics, particularly focusing on reduction in muscle activation levels as the primary indicator of the efficacy of the BSE [9]. In dynamic motor tasks, however, reduced muscle activity necessarily equates to neither lower muscle force nor reduced spinal loads [10]. Furthermore, such simplistic evaluations may overlook drawbacks such as increased discomfort and movement restriction, which can compromise both user experience and safety, ultimately limiting the practical application of BSEs [11,12]. Accordingly, a multifaceted approach has been emphasized [13], and a recent study has integrated objective physiological and subjective assessments to show the efficacy of a BSE [14].

Although the efficacy of BSEs has been comprehensively studied in controlled laboratory settings, real-world evaluations remain limited and have largely been based on subjective assessments due to challenges in collecting high-quality sensor data. A four-week study on automobile manufacturing workplaces included evaluation of user discomfort, usability, and acceptance on the Laevo exoskeleton [15]. Another study investigated work intensity and musculoskeletal discomfort throughout the 24-weeks in field intervention among logistic-workers and found overall decrease in work intensity [16,17]. These long-term investigations on user perceptions may reflect the feasibility of adopting BSEs to the workplaces, but quantitative biomechanical efficacy of BSEs has not been clarified yet in such studies.

Examining the correlation among biomechanical and subjective measures may help address the limited set of accessible variables in real-world settings. Although a few earlier studies explored correlation between composite measures of muscle activation and estimated joint forces [10,18], these efforts were limited to objective biomechanical outcomes, which are often difficult to obtain in field-based evaluations. In the context of shoulder assist exoskeletons, previous study investigated the relationship between user satisfactions and biomechanical outcomes, and found significant correlation between range of motions in the neck and shoulder and user satisfaction, suggesting possible estimation of satisfaction from changes in joint range of motions [19]. However, in the domain of BSEs, studies not only lack comprehensive analysis of correlation between biomechanical variables, but also rarely examine their associations with subjective assessments. Considering the practical utility of subjective assessments in real-world settings, identifying their correlation with objective biomechanical metrics is crucial for meaningful interpretation and application of BSEs.

In this study, we aim to evaluate both the biomechanical and subjective effects of a previously developed passive BSE, Angel X, during a manual load handling task, and to explore correlation among the measurements. Specifically, we used musculoskeletal simulations driven by experimentally measured ground reaction forces (GRFs) to estimate L5–S1 joint reaction forces and modeled the contact forces between the BSE and human body. Furthermore, we investigated the relationships among various biomechanical and subjective metrics, including L5–S1 joint reaction forces, muscle activation, perceived exertion, and perceived discomfort.

## Methods

### Participants

Data from twelve healthy young males (age: 23.8 ± 2.8 years; height: 177 ± 4.1 cm; weight: 73.1 ± 9.2 kg) were included. The required sample size was determined using G-power software [20]. Based on a large effect size of 0.8, a statistical significance level of 0.05, and a desired power of 80%, a minimum of 12 participants was required. We recruited 15 participants to account for potential dropouts. The final analysis included 12 participants as three were excluded due to contaminated GRFs. The participants had no history of musculoskeletal, orthopedic, and cardiovascular disorders. All participants demonstrated their ability to lift a 15 kg weight box during the acclimatization period. This study was conducted with approval from the institutional review board (IRB) of Seoul National University (IRB No. 2303/003–009) and adhered to the principles outlined in the Declaration of Helsinki. All participants provided informed written consent before participating in the study. The recruitment period for this study was from 28 June 2023–18 July 2023.

### Equipment

#### Passive back support exoskeleton and weight box.

The passive back support exoskeleton (BSE) evaluated in this study was the Angel X (Angel Robotics Co., Ltd.). The device is designed to reduce the wearer’s biomechanical load during lifting tasks involving spinal flexion and extension. As a passive system without powered actuators, it assists lifting by storing elastic energy during trunk flexion and returning it during extension, thereby generating a flexion-dependent restoring torque that can partially counteract the gravitational flexion moment from the trunk and external load. To generate assistive joint torques in a physiologically consistent manner, the Angel X employs bilateral spring–cam mechanisms at the hips; the cam profile is engineered to reproduce the desired torque–angle relationship. The maximum assistive torque of each spring–cam module was designed to be adjustable between 14 and 16 Nm and was set to the maximum level in this study. The measured torque from the actual used module reached 17.7 Nm during flexion at 68° of flexion, whereas the torque during extension was 14.8 Nm at the same angle, reflecting the hysteresis effect (Fig 2). The device weighs 3.0 kg and permits 120° hip flexion, 25° extension, 30° adduction, 90° abduction, and 50° axial rotation. Detailed specifications can also be found in our previous study [14].

A lightweight box capable of securely holding a 15-kg load was used for the lifting tasks. The load magnitude was selected with reference to established industrial load-handling guidelines [21], which categorize this range as spanning mild to high task intensity.

### Experimental procedure

The experimental procedure for the study is detailed in our previous work [14]. To briefly recap, participants were fitted with the BSE prior to the first trial. Participants were given at least 10 minutes to familiarize themselves with lifting and lowering tasks while wearing the BSE. After acclimatization, we attached twelve surface electromyography (EMG) sensors (8 Trigno Avanti and 4 Trigno Mini, Delsys, Natick, MA, USA) to the target muscles following SENIAM guidelines [22]. Considering the most frequently investigated target muscles in previous studies [13], we utilized only 4 EMG sensors which were bilaterally attached to the thoracic erector spinae (TES) and lumbar erector spinae (LES) muscles as shown in Fig 1A. Nine infra-red cameras (Arqus A5, Qualisys AB, Sweden) were used to record the three-dimensional kinematic data at a sampling frequency of 100 Hz. We modified OpenSim model of lifting full-body (LFB) model and placed 49 reflective markers bilaterally on the anatomical landmarks (Fig 1A). We additionally attached two markers to the top side of the box, whose vertical movement was later used as references for identifying the initiation and termination of the lifting and lowering phases.

**Fig 1:**
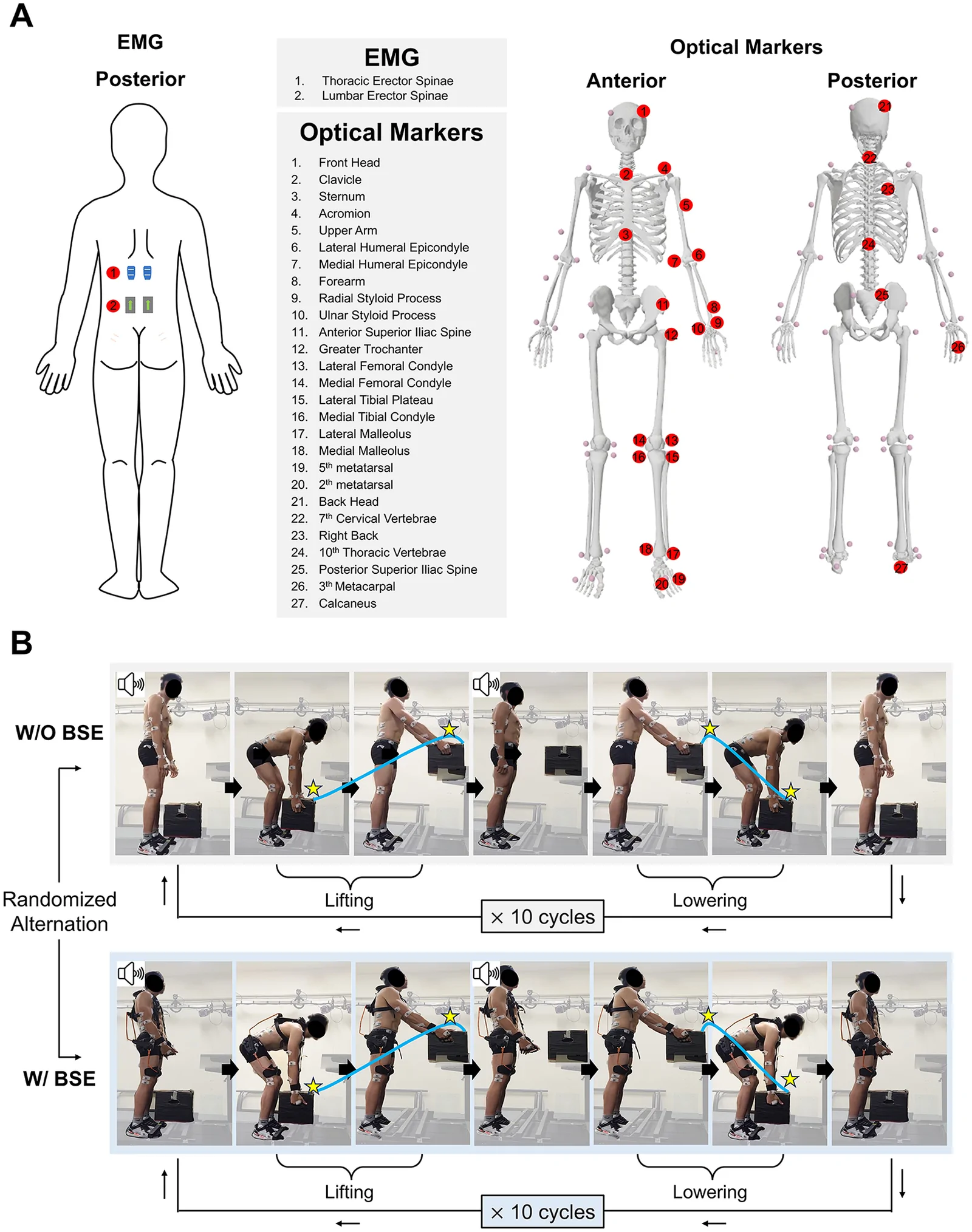
Overview of sensor placements and lifting task protocol. (A) EMG and optical marker placements for data collection. (B) Experimental lifting task performed with and without the BSE in a randomized order. The blue solid line represents the marker trajectory, and the stars indicate the start and end points of the lifting and lowering phases. The speaker symbol indicates the timing of the acoustic cues, which were provided at 5 s intervals. Reprinted from [14] under a CC BY 4.0 license, with permission from Springer Nature Customer Service Center GmbH, original copyright 2025.

Fig 1B demonstrates the experimental protocol. It included six blocks of alternating with-device (W/) and without-device (W/O) conditions where the initial condition was randomly assigned. Each block consisted of 10 cycles of lifting a 15 kg load from ankle height to a table at waist height and lowering it back down. We used a consistent 12 bpm acoustic cue to standardize the timing of the start of each lifting and lowering movement. The start of the lifting phase was defined as the time point at which the marker on the box began to move vertically, and the end was defined as the time point at which the marker reached its local maximum vertical position. The lowering phase was defined from the local maximum vertical position of the marker to the time point at which its vertical movement stopped. The start and end timing of each lifting and lowering phase are denoted by stars in Fig 1. Participants rated their whole-body rate of perceived exertion (RPE) and local perceived discomfort (LPD) after each block during a 5-minute rest period.

### Data processing

#### EMG signal.

We recorded EMG signals at a sampling frequency of 2148 Hz and then resampled to 1000 Hz. To remove any movement related artifact noise, a fourth-order Butterworth band-pass filter with a range of 20–400 Hz was applied [23]. The full-wave rectified signals were subsequently smoothed with a fourth-order Butterworth low-pass filter with a cut-off frequency set at 2.7 Hz [24]. Average muscle activation was then derived from the root mean square (RMS) of the preprocessed EMG signal, and muscle effort was calculated using integrated EMG (iEMG) over the corresponding time interval, with both measures calculated separately for the lifting and lowering phases. Thus, taking a single trial from one of the three W/O condition blocks as an example, 10 RMS values and 10 iEMG values were obtained for each of the lifting and lowering phases.

The use of the BSE could slightly alter the execution time of each lifting and lowering phase. Therefore, to account for muscle activity in terms of total command effort [25], we additionally selected iEMG along with EMG RMS. Because load handling is a dynamic task in which muscle activity can surpass the peak maximum voluntary contraction, we used reference voluntary contraction (RVC) for normalization based on a previous study [26]. RVC for EMG RMS was defined as the maximum value of the obtained RMS values during the whole experimental trials and used as the normalization reference for each participant. For iEMG, the normalization reference was defined as the maximum value of the calculated iEMG values during the whole trials for each participant. The initiation of data collection was synchronized to the motion capture system using a sync box.

#### Musculoskeletal simulation framework.

The raw coordinates of the 51 reflective markers (49 anatomical markers and 2 on the box) were filtered using a fourth-order Butterworth low-pass filter with a 6 Hz cut-off frequency. GRF data were collected at a sampling frequency of 1000 Hz on a force plate-embedded treadmill (Instrumented treadmill, Bertec Inc., Columbus, OH, USA). The force data were filtered using a second-order Butterworth low-pass filter with a 10 Hz cut-off frequency. The synchronization of force data collection with the motion capture system was managed via motion capture software.

We utilized OpenSim musculoskeletal simulation [27] for the estimation of L5–S1 joint load. The used LFB model was previously validated against actual EMG measurements of torso muscle during two-handed stoop and squat lifting tasks [28]. Each hand’s mass was increased by half of the load mass to model external forces exerted by the load on the hands during the lifting and lowering phases [29]. In the W/ condition, the three main components of the BSE were attached to the musculoskeletal model at predefined contact points on the torso, pelvis, and thigh as welded joints (Fig 2). The mass and moment of inertia were derived from the CAD model of the BSE. We modeled the contact force at the chest, hip, and thigh on the musculoskeletal model based on the measured torque profile from the waist support module. For each time frame of the simulation, the assistive torque corresponding to the measured trunk flexion angle was determined from the torque–angle profile (Fig 2). The required contact forces were then calculated using the moment arm between the waist support module and the predefined contact points on the sternum and thigh, such that the resulting moment matched the torque from the measured profile.

**Fig 2:**
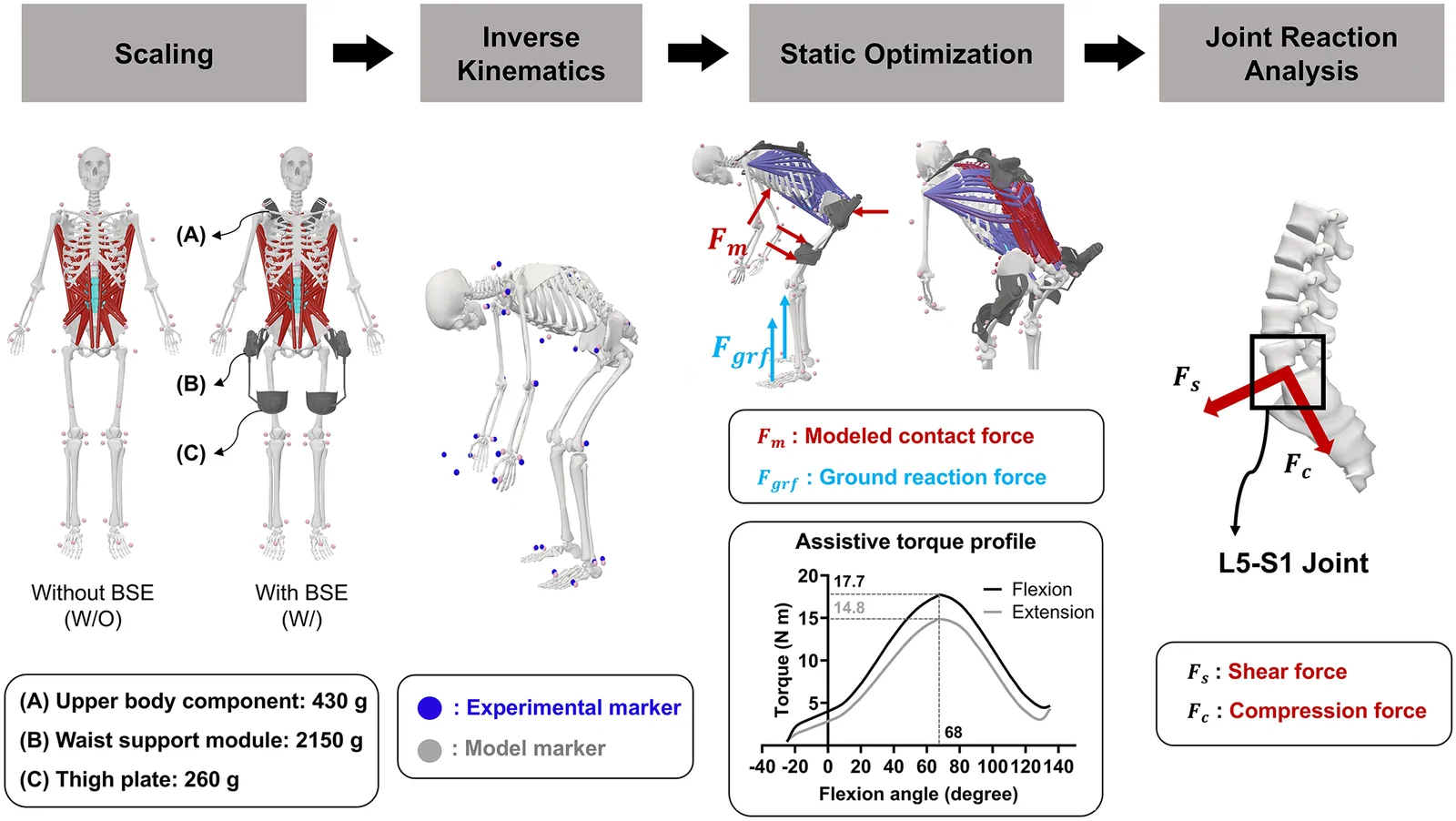
Illustration of the musculoskeletal simulation procedure with assistive torque profile.

The musculoskeletal simulation consisted of model scaling, inverse kinematics, static optimization, and joint reaction analysis in OpenSim 4.4. First, the LFB model was scaled to match each participant’s body mass and static marker positions using AddBiomechanics [30]. Next, inverse kinematics was performed to compute joint angles based on the collected three-dimensional marker trajectories. We then estimated muscle forces through static optimization using joint kinematics and measured external forces. Finally, joint reaction analysis was conducted to calculate compression and shear forces on the L5–S1 joint. The simulation was performed for each participant under W/ and W/O conditions, and for each lifting and lowering phases, which resulted in 30 lifting and lowering phases per condition. Profiles of L5–S1 joint compression and shear forces were represented as averaged value for each lifting phase and condition.

#### Subjective assessments.

Participants assessed their whole-body rate of perceived exertion (RPE) using the Borg CR-10 scale [31] which ranges from 0 (no exertion) to 10 (maximum exertion). We assessed musculoskeletal discomfort in the lower back, specifically local perceived discomfort (LPD), which is characterized by sensations of stiffness, pain, and localized fatigue [32]. The ratings of assessments were averaged across trials under the same condition.

### Statistical analysis

Outcome measures of muscle activation, joint load, subjective assessments between the two experimental conditions of 12 participants were compared using paired t-tests after verifying the normality assumption with the Shapiro-Wilk test. When the normality assumption was violated, the non-parametric Wilcoxon signed-rank test was used. The effect sizes of the intervention were calculated using Cohen's d. We performed Pearson’s correlation analysis to evaluate the linear relationship between changes in resultant measures, and Spearman correlation analysis was conducted when the normality assumption was not met. To control for Type I error inflation due to multiple comparisons, p-values were adjusted within muscle activation, L5–S1 joint load, and subjective assessment outcomes using the Benjamini–Hochberg false discovery rate procedure. All statistical analyses were conducted using jamovi 2.6.23 software [33], and the Benjamini–Hochberg false discovery rate correction was performed using MATLAB, with the statistical significance level (α) set at 0.05.

## Results

### Muscle activation

Fig 3 presents the mean and standard deviation (SD) of RMS and iEMG for erector spinae muscle activation in 12 participants under the two experimental conditions. A paired t-test showed that the BSE significantly decreased the EMG RMS of the TES muscle during the lifting (p = 0.005, Cohen’s d = 1.183, 95% CI [0.4209, 1.916]) and the lowering phase (p < 0.001, Cohen’s d = 1.935, 95% CI [0.9426, 2.898]). For the LES muscle, the EMG RMS showed no statistical difference between the two experimental conditions during the lifting phase. In contrast, in the lowering phase, the BSE significantly decreased the EMG RMS (p = 0.002, Cohen’s d = 1.385, 95% CI [0.5658, 2.174]).

**Fig 3:**
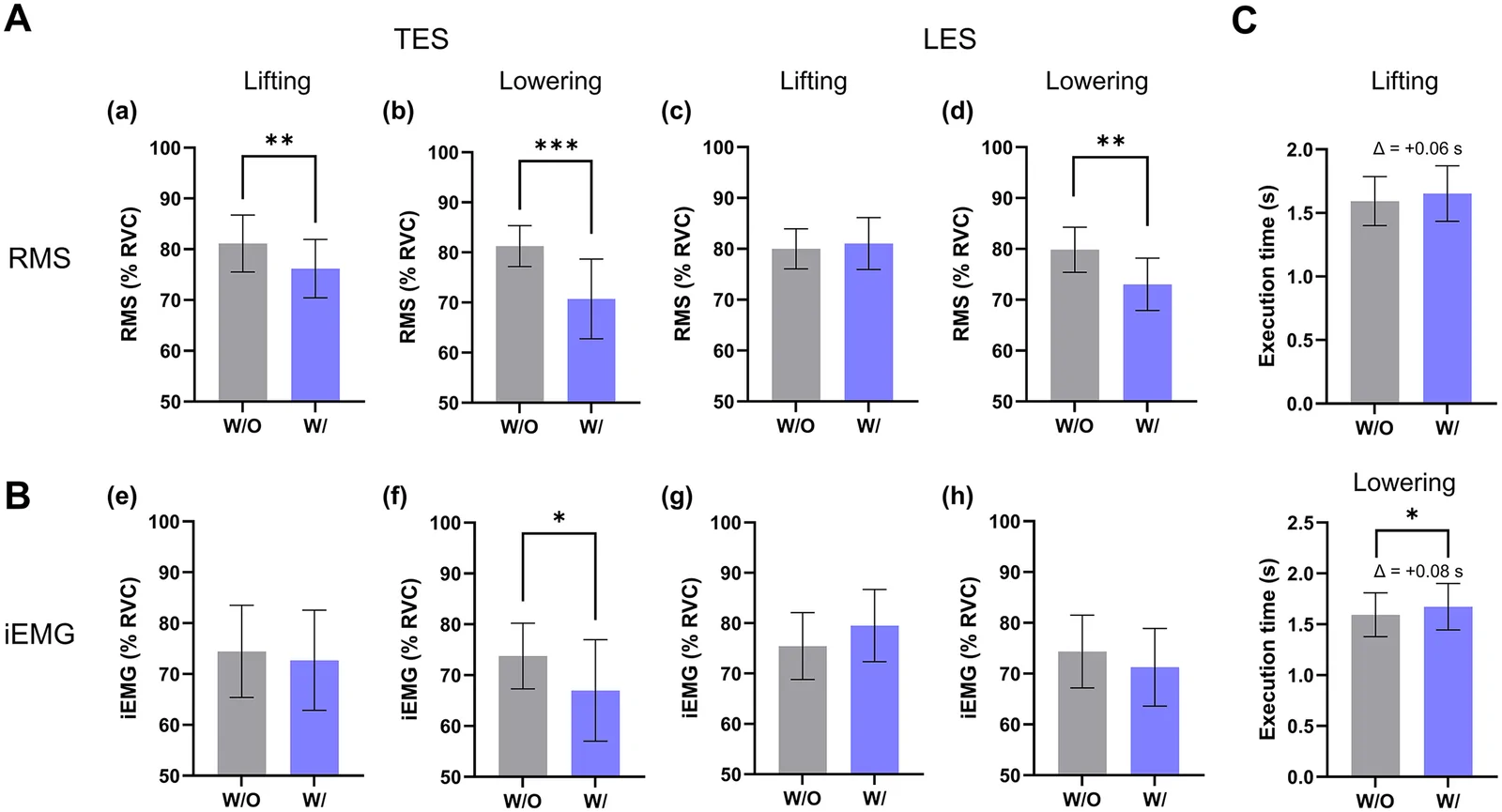
Changes in EMG RMS and iEMG of the TES and LES muscles. (A) Mean and standard deviation bars of the EMG RMS of TES and LES muscles in 12 participants during the lifting and lowering phases under the two experimental conditions (W/O: without the BSE, W/: with the BSE). (B) Mean and standard deviation bars of the iEMG of the TES and LES muscles in 12 participants during the lifting and lowering phases under the two conditions. (C) Mean and standard deviation of execution time for the lifting and lowering phases in 12 participants under the two conditions. The asterisks indicate statistical significance (*p < 0.05, **p < 0.01, ***p < 0.001).

The effort of the erector spinae muscles was also investigated from iEMG (Fig 3B). The BSE did not significantly reduce the iEMG in the TES muscle during lifting but showed a significant reduction during lowering (p = 0.01, Cohen’s d = 0.99, 95% CI [0.2772, 1.673]). The statistical analysis revealed that the BSE did not significantly affect LES iEMG during either lifting or lowering.

### L5–S1 joint load

Fig 4 shows the mean and SD of the L5–S1 joint load for 12 participants under the two experimental conditions. Pair-wise comparisons revealed that the BSE significantly reduced the compression force during the lifting phase from 53.0 N/kg to 49.0 N/kg (p < 0.001, Cohen’s d = 1.575, 95% CI [0.6984, 2.421]) and during the lowering phase from 50.6 N/kg to 47.7 N/kg (p < 0.001, Cohen’s d = 1.522, 95% CI [0.6617, 2.352]) (Fig 4A). Fig 4B shows that the BSE induced a greater decrease in the shear force, reducing it from 11.8 N/kg to 6.5 N/kg during lifting (p < 0.001, Cohen’s d = 9.029, 95% CI [5.2696, 12.638]) and from 11.3 N/kg to 6.2 N/kg during lowering (p < 0.001, Cohen’s d = 8.819, 95% CI [5.1450, 12.347]).

**Fig 4:**
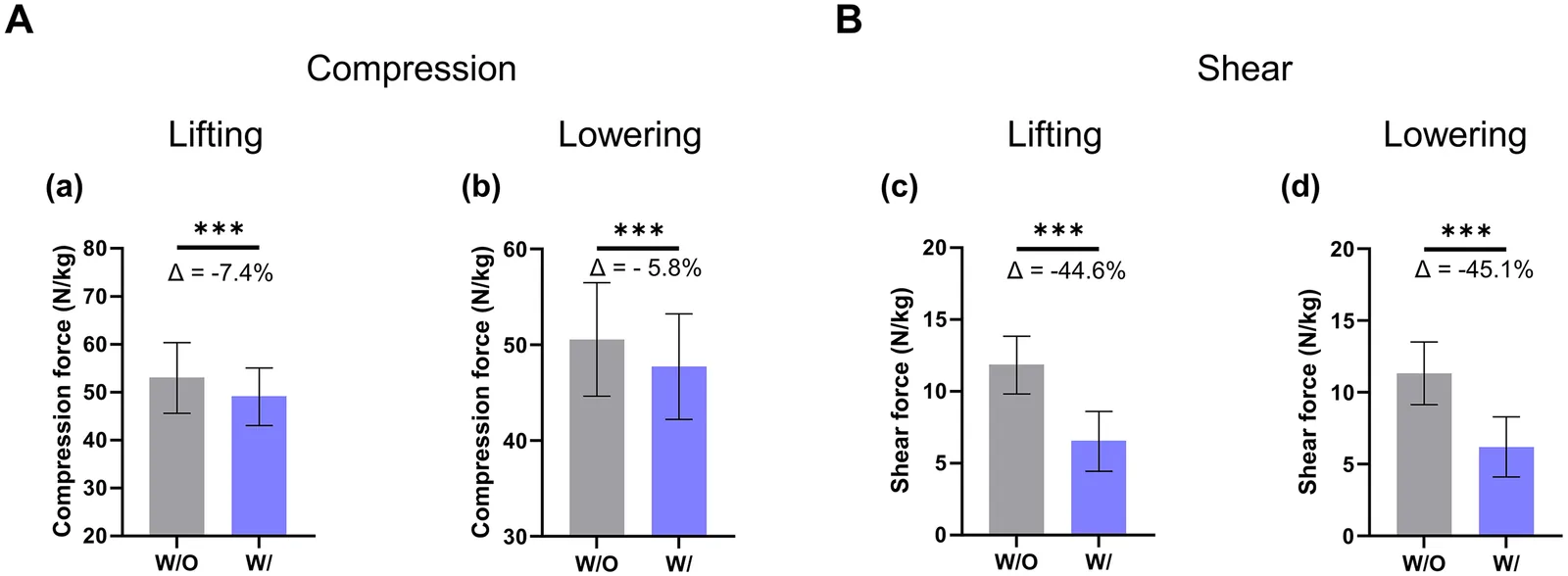
Changes in compression and shear forces in the L5–S1 joint. (**A**) Mean and standard deviation bars of the compression force in the L5–S1 joint in 12 participants during the lifting and lowering phases under the two experimental conditions (W/O: without the BSE, W/: with the BSE). (**B**) Mean and standard deviation bars of the shear force in the L5–S1 joint in 12 participants during the lifting and lowering phases under the two conditions. The asterisks indicate statistical significance (***p < 0.001).

### Subjective assessments

The mean and SD of RPE and LPD for 12 participants under the two conditions are shown in Fig 5A and Fig 5B, respectively. A significant main effect of the BSE was revealed for RPE (p = 0.019, Cohen’s d = 0.903, 95% CI [0.2116, 1.567]). LPD decreased by an average of approximately 22%, but the reduction was not statistically significant (p = 0.272, Cohen’s d = 0.334, 95% CI [−0.2553, 0.909]).

**Fig 5:**
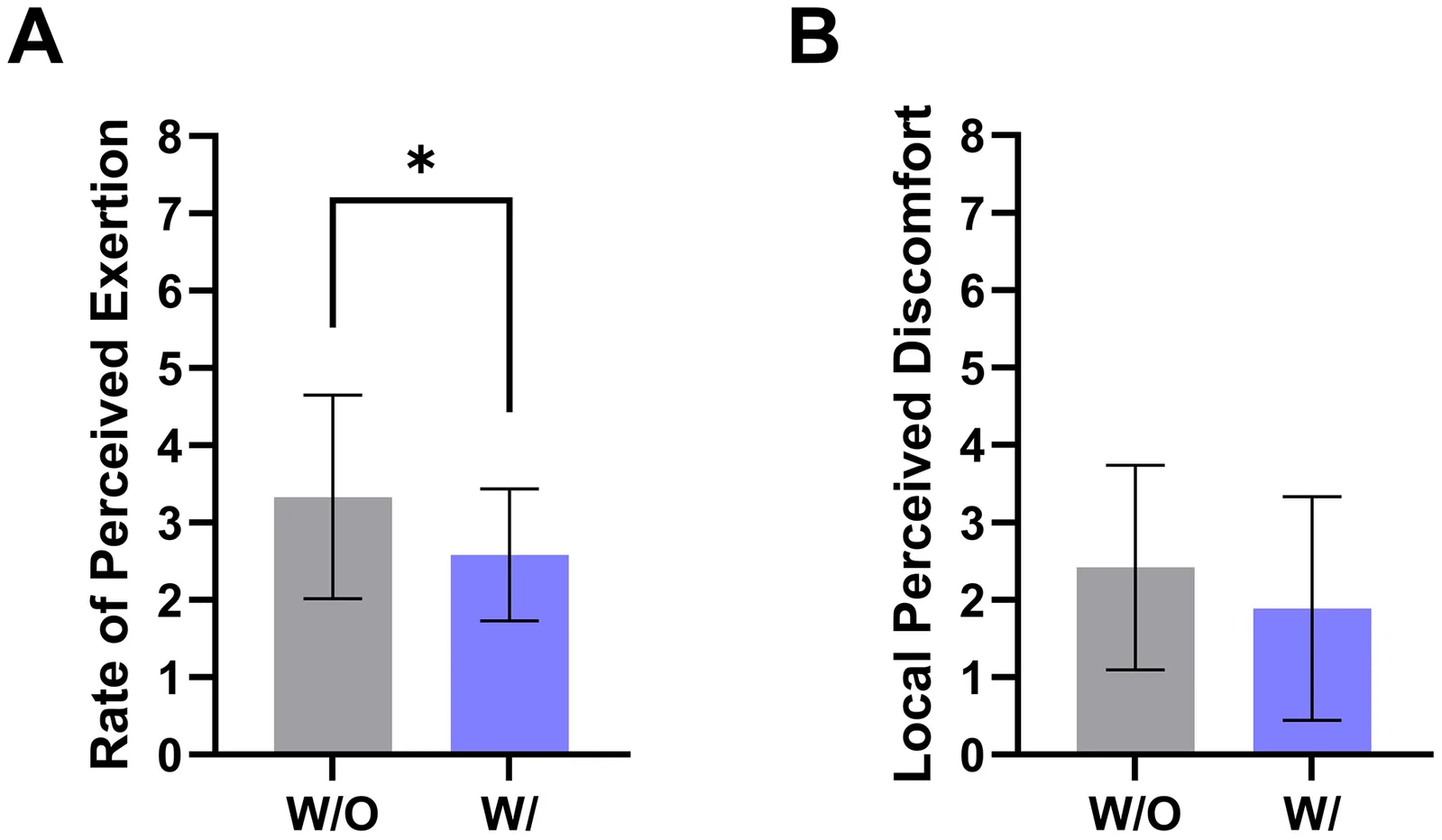
Changes in RPE and LPD. (A) Mean and standard deviation bars of the RPE in 12 participants under the two experimental conditions (W/O: without the BSE, W/: with the BSE). (B) Mean and standard deviation bars of the LPD in 12 participants under the two conditions. The asterisks indicate statistical significance (*p < 0.05).

### Correlation between L5–S1 joint load, muscle activation, and subjective assessments

Table 1 shows the results of the correlation analysis between changes in L5–S1 joint load (shear and compression) and changes in muscle activation outcome (EMG RMS) during the lifting and lowering phases for 12 participants. The analysis revealed no correlation between the joint load and EMG outcome measures.

**Table 1:** The results of the Pearson’s correlation analysis between changes in L5–S1 joint load and changes in muscle activation outcome measure.

Phase	L5–S1 Joint load	TES RMS	LES RMS
Lifting	Shear	R^2^ = 0.041 r = −0.203 p = 0.777 95% CI [−0.696, 0.419]	R^2^ = 0.370 r = 0.608 p = 0.287 95% CI [0.053, 0.876]
	Compression	R^2^ = 0.042 r = 0.205 p = 0.777 95% CI [−0.418, 0.697]	R^2^ = 0.028 r = −0.168 p = 0.777 95% CI [−0.677, 0.449]
Lowering	Shear	R^2^ = 0.056 r = 0.236 p = 0.777 95% CI [−0.391, 0.713]	R^2^ = 0.181 r = 0.426 p = 0.668 95% CI [−0.195, 0.804]
	Compression	R^2^ = 0.000 r = 0.018 p = 0.956 95% CI [−0.562, 0.586]	R^2^ = 0.018 r = −0.133 p = 0.777 95% CI [−0.657, 0.477]

Table 2 shows the correlation between changes in subjective assessments and changes in outcome measures of L5–S1 joint load and erector spinae muscle activation. The statistical analysis revealed no significant correlation between any of the measures.

**Table 2:** The results of the Pearson’s correlation analysis between changes in subjective assessments and changes in outcome measures of L5–S1 joint load and muscle activation.

Subjective assessments	L5–S1 Joint load		TES		LES	
	Shear	Compression	RMS	iEMG	RMS	iEMG
RPE	R^2^ = 0.003 r = 0.057 p = 0.859 95% CI [-0.534, 0.611]	R^2^ = 0.071 r = 0.267 p = 0.687 95% CI [-0.363, 0.729]	R^2^ = 0.135 r = −0.368 p = 0.687 95% CI [-0.778, 0.261]	R^2^ = 0.062 r = −0.249 p = 0.687 95% CI [-0.720, 0.379]	R^2^ = 0.219 r = −0.468 p = 0.687 95% CI [-0.821, 0.145]	R^2^ = 0.056 r = −0.237 p = 0.687 95% CI [-0.714, 0.390]
LPD	R^2^ = 0.019 r = −0.139 p = 0.859 95% CI [-0.660, 0.473]	R^2^ = 0.003 r = −0.058 p = 0.859 95% CI [-0.611, 0.534]	R^2^ = 0.225 r = −0.474 p = 0.687 95% CI [-0.824, 0.137]	R^2^ = 0.080 r = −0.283 p = 0.687 95% CI [-0.737, 0.347]	R^2^ = 0.123 r = −0.350 p = 0.687 95% CI [-0.769, 0.280]	R^2^ = 0.007 r = −0.083 p = 0.859 95% CI [-0.627, 0.515]

## Discussion

In this study, we investigated the effects of a passive BSE on biomechanical load during the manual load handling task, and explored the correlation among muscle activation, estimated joint load, and subjective assessments. We found that the BSE lowered muscle activation level, joint reaction force on the lower back, and perceived work intensity. However, no measures were significantly correlated.

We analyzed the average muscle activity of major trunk extensor muscles during lifting and lowering and found that the BSE reduced the EMG RMS in the TES muscles in both phases and in the LES muscles during the lowering phase. Reductions in trunk extensor muscle activation align with the results of multiple earlier studies, as outlined in a systematic review [13,34]. The greater effect observed during lowering compared to lifting may be primarily attributed to the inherent hysteresis effect of the spring–cam module. As described in the Methods, the measured peak torque from a single module during flexion was approximately 20% greater than that during extension (17.7 Nm vs. 14.8 Nm). This larger assistive torque may have reduced the required muscular effort by reducing the required biological torque to generate trunk movement. Additionally, muscle dynamics associated with generating larger force during eccentric contractions may have contributed to the greater reduction in muscle activation during lowering, as discussed in our previous work [14]. We also found that muscle effort computed using iEMG showed an overall smaller effect in the W/ condition compared with EMG RMS (Fig 3A, B). iEMG did not show significant differences in the TES during lifting (Fig 3B(e)) or in the LES during lifting (Fig 3B(g)) and lowering (Fig 3B(h)). Because iEMG accounts for activation duration, the increased execution time in the W/ condition, which was slight during lifting and significant during lowering (Fig 3C), may explain the discrepancy between the EMG RMS and iEMG results.

We estimated L5–S1 joint reaction force through the joint reaction analysis from musculoskeletal simulation. The result indicates that the BSE decreased the compression and shear forces during lifting and lowering with statistical significance, and the reduction was much more notable in shear direction (Fig 4). The assistive torque generated by the BSE is transmitted as a vertical contact force on the chest, which directly counteracts the anterior shear load produced by gravity acting on the trunk. This mechanical support is particularly effective in reducing shear force at the L5–S1 joint, as it counteracts the gravitational force. The reduction in compression force is primarily mediated by the decreased demand on the trunk extensor muscles. These muscles are the main source of the extension moment required to lift or lower the trunk and simultaneously a major contributor to compressive loading at the L5–S1 joint [35]. The BSE’s assistance in trunk extension reduces the necessary muscle force, thereby leading to a corresponding decrease in compression load.

Our findings are consistent with prior studies. For example, a previous study using Paexo Back reported a 20% reduction in L5–S1 joint compression force when wearing the exoskeleton [36]. Similarly, Madinei et al. reported significant reductions in peak compression and shear forces at the L5–S1 joint [10]. Differences in the magnitude of reduction with earlier studies may be attributed to variations in the mechanical design of the supportive torque profile. Unlike these previous studies, however, we experimentally measured GRFs rather than estimating them to enhance the accuracy of the L5–S1 joint reaction force estimates and strengthen the correlation analysis between these estimates and other biomechanical outcomes. Although our musculoskeletal model does not account for co-contraction of torso muscles, which is known to increase joint load and contribute to spinal stability [37,38], the previous validation of this musculoskeletal model [28] and three-dimensionality of the model [39] ensures good agreement with muscle coactivity. Therefore, the consistent application of the methodology ensures the internal validity of the present findings.

Despite the BSE’s biomechanical benefits, its overall effectiveness may be diminished if these advantages do not translate into improved user’s perceived effort or comfort. In this study, the participants reported a significantly lower level of perceived exertion in a highly repetitive task as shown in Fig 5A. The acute effect found in this study aligns with previous findings on exoskeleton use [40,41]. In contrast, we found no statistically significant reduction of LPD (Fig 5B). One previous study showed decreased LPD in lower back during static forward bending [42], and another study reported that BSEs had main effects on reducing LPD [41]. In contrast, other studies showed no efficacy of the investigated BSE in reducing perceived discomfort in dynamic task [15,43], and even increased discomfort in chest region was observed [15]. Certain exoskeleton designs have been reported to cause discomfort due to added weight or restricted mobility, which can lead to decreased performance and increased perceived task difficulty [13]. Therefore, as subjective perceptions do not solely rely on mechanical benefits provided by BSEs, it is reasonable that interpretation of subjective assessments should be confined to specific BSE, task, and targeted group. As our previous results demonstrated a significantly lower LPD in a prolonged lifting task where the BSE delayed muscular fatigue [14], the non-significant acute effect observed in this study may result in long-term benefits when applied in real-world settings. However, this must be clarified upon a field-based study.

We further explored correlation among outcome measures of biomechanical and subjective assessments. Table 1 shows the relationships between changes in estimated L5–S1 joint load and changes in EMG RMS of the erector spinae muscles. No significant correlation was found between changes in L5–S1 joint loads and changes in EMG RMS of the erector spinae muscles. A previous study also reported no significant correlation between changes in L5–S1 shear force and muscle activity of trunk extensor and total trunk muscles during lifting and lowering in the male group [10], which aligns with our finding.

Considering that muscle force is a major source of accounting for joint load, the lack of linear correlation may mainly be attributed to the nonlinearity between muscle activity and muscle forces. One possible explanation is the flexion-relaxation phenomenon, in which lumbar extensor muscle activity decreases as passive tissues increase their contribution to supporting the trunk [44]. However, the redistribution of loads between active and passive structures during BSE use remains unclear. Furthermore, the extent of load sharing between active and passive structures can vary across individuals, even at similar trunk postures [44]. Therefore, subtle changes in trunk kinematics induced by BSE use may have further complicated the relationship between muscle activity and L5–S1 joint load. Another possible explanation is individual differences in trunk muscle activation strategies during the task. The same joint kinematics and kinetics can be achieved with different patterns of muscle activation due to muscle redundancy, which may affect erector spinae muscles activation without being fully reflected in joint load estimates from musculoskeletal simulations. Collectively, these factors may have obscured a simple linear relationship between changes in EMG RMS and changes in L5–S1 joint loads, suggesting that erector spinae EMG alone is insufficient to estimate changes in spinal loading during BSE use.

In addition, we also found no significant correlation between changes in subjective assessments and outcome measures of L5–S1 joint load and muscle activation in erector spinae muscles (Table 2). A previous study found a decrease in RPE after long-term intervention of a BSE and suggested that the reduction was likely driven by decreased muscle activity [16]. However, since no significant relationship was found between the two in our study, the primary cause of the reduced perceived work intensity may not have been the change in muscle activation level. Our finding also clarifies the limitation of the evaluation which solely relies on user perception. While user feedback in field-based studies is crucial for confirming perceived efficacy, our results suggest that relying only on subjective measures may lead to misleading conclusions about the biomechanical effectiveness of BSEs in reducing spinal load or muscle activation.

Multiple limitations of our study need to be clarified. First, we recruited only healthy young males, which limits generalizability of our findings. Although females can also be employed as industrial workers, we considered that majority of the workers are still males, and the accuracy of musculoskeletal modeling-based analysis has been mostly validated for males [28,45]. Second, the manual lifting task used in this study does not fully represent the wide variety of tasks performed in typical workplaces. Similarly, subjective assessments obtained during this specific experimental protocol may not fully reflect long-term perceptions during real work. In this study, we selected representative movements under a controlled laboratory setting to rigorously evaluate the relationships among biomechanical outcomes. Future studies should include field-based evaluations in actual workplace environments. Third, while the mass and inertia of the BSE were incorporated by attaching its segments to the musculoskeletal body, the contact forces from the BSE were modeled using static dynamics. This approach may have resulted in inaccuracies in estimating the actual contact forces. However, dynamic effects of both translational and rotational acceleration are likely negligible since the BSE's mass was minimal relative to the participants’ body mass, and the task was performed at a relatively slow pace using periodic acoustic cues. Lastly, this study primarily focused on the lumbar region and did not account for the BSE’s effects on other body regions, such as the knees or abdomen. Passive devices function by storing and releasing elastic potential energy through mechanical components, inherently redistributing force through contact with multiple body segments. This undesirable contact may constrain movement or cause discomfort in non-target areas. However, we previously observed a significant reduction in discomfort in the arm and thigh [14], suggesting that the BSE’s negative impact on other body regions may be minimal.

## Conclusion

In this study, we conducted biomechanical and subjective evaluations on the efficacy of the BSE, Angel X, and investigated correlation between examined measurements. The BSE reduced muscle effort and provided significant assistance in reducing the joint load on the lower back which was revealed by musculoskeletal simulation approach. Furthermore, participants reported that the BSE decreased perceived work intensity. However, significant correlation was not found between investigated measures. The results highlight that each metric of biomechanical and subjective evaluations captures a distinct aspect of BSE efficacy, and that one cannot be generally inferred from other metrics.
